# The geographic mosaic of herbicide resistance evolution in the common morning glory, *Ipomoea purpurea*: Evidence for resistance hotspots and low genetic differentiation across the landscape

**DOI:** 10.1111/eva.12290

**Published:** 2015-08-13

**Authors:** Adam Kuester, Shu-Mei Chang, Regina S Baucom

**Affiliations:** 1 Department of Ecology and Evolutionary Biology, 830 North University, University of MichiganAnn Arbor, MI, USA; 2 Plant Biology Department, University of GeorgiaAthens, GA, USA

**Keywords:** approximate Bayesian computation, glyphosate, *Ipomoea purpurea*, morning glory, resistance, SSR, weed

## Abstract

Strong human-mediated selection *via* herbicide application in agroecosystems has repeatedly led to the evolution of resistance in weedy plants. Although resistance can occur among separate populations of a species across the landscape, the spatial scale of resistance in many weeds is often left unexamined. We assessed the potential that resistance to the herbicide glyphosate in the agricultural weed *Ipomoea purpurea* has evolved independently multiple times across its North American range. We examined both adaptive and neutral genetic variations in 44 populations of *I. purpurea* by pairing a replicated dose–response greenhouse experiment with SSR genotyping of experimental individuals. We uncovered a mosaic pattern of resistance across the landscape, with some populations exhibiting high-survival postherbicide and other populations showing high death. SSR genotyping revealed little evidence of isolation by distance and very little neutral genetic structure associated with geography. An approximate Bayesian computation (ABC) analysis uncovered evidence for migration and admixture among populations before the widespread use of glyphosate rather than the very recent contemporary gene flow. The pattern of adaptive and neutral genetic variations indicates that resistance in this mixed-mating weed species appears to have evolved in independent hotspots rather than through transmission of resistance alleles across the landscape.

## Introduction

The evolution of herbicide resistance in weedy plants is an excellent example of adaptation to strong human-mediated selection (Vigueira et al. 2013), and one that, like other examples of resistance to xenobiotics, carries an immense ecological and economic cost. Over 230 cases of resistance have evolved in the relatively short period of time in which herbicides have been utilized for weed control (Heap 2014). This resistance evolution, in practical terms, can mean a dramatic loss of efficacy of weed control for large areas of land, as well as a concomitant change in the weed populations inhabiting both crop fields and crop margins (Culpepper 2006; Webster and Nichols 2012). In addition to providing novel study systems for rapid evolutionary change, examination of the forces underlying the evolution of herbicide resistance across populations is a key to developing strategies for reducing their impact—one that is estimated to be as high as 33 billion USD, yearly (Pimentel et al. 2005).

The ability of a population to adapt to the strong selection from herbicide application is ultimately dependent on the amount and type of genetic variation that is available to selection (Jasieniuk et al. 1996; Delye et al. 2013). Thus, the population size, genetic architecture, standing genetic variation, amount of gene flow, and the mutation rate (Hedrick et al. 1976) are all interacting factors that dictate the emergence of resistance across populations. While it has been hypothesized that gene flow between populations may be a more likely cause than novel mutations for the appearance of resistance across the landscape (Jasieniuk et al. 1996), of the few species that have been investigated, only highly self-pollinating species exhibit isolation by distance in the level of resistance—a pattern consistent with the idea that gene flow contributes to the spread of resistance alleles (Osuna et al. 2011; Okada et al. 2013). In comparison, the outcrossing grass species *Alopecurus myosuroides* exhibits a mosaic resistance pattern and no evidence of isolation by distance across populations, suggesting that resistance has evolved independently on a local scale (Delye et al. 2010). Unfortunately, because the majority of herbicide resistance studies are generally either descriptive examinations of the level of resistance across an often limited number of natural populations (Beckie et al. 2000; Preston and Powles 2002; Neve and Powles 2005; Bernards et al. 2012), or investigations of the molecular basis of resistance (Marshall and Moss 2008; Cseh et al. 2009; Beckie et al. 2011; Lang et al. 2011; Sada et al. 2013), we currently have a very limited view of how within- and between-population processes, such as gene flow and heterogeneous selection can influence herbicide resistance evolution across the landscape. Such examinations are, to our knowledge, lacking in species that employ mixed-mating systems even though ∼32% of weedy plants exhibit a mixed-mating strategy (Kuester et al. 2014). Thus, in addition to the need for more examinations of resistance evolution across the landscape, we also have a broad gap in our understanding of the spatial context of resistance evolution in species that are predominately insect pollinated and/or exhibit mixed mating.

The study of the spatial scale of herbicide resistance is relevant to both basic evolutionary biologists and applied scientists for somewhat disparate reasons: Evolutionary researchers are fascinated by the repeatability of the evolutionary process whereas applied scientists, who want to maintain low levels of resistance in nature, need to understand where control efforts are best implemented. For example, different management recommendations would be made if resistance evolved in a single population and spread compared to a scenario where herbicide resistance evolved independently in separate populations. To determine which scenario is most likely for a given weed species, researchers generally pair an assessment of the level of resistance across populations collected from the landscape, often in a common garden study, with an examination of the pattern of neutral genetic variation across these same populations. If, for example, the level of resistance displays a pattern of isolation by distance, one can infer that resistance is spread by gene flow either on a local scale or at greater distances; an assessment of neutral genetic variation that likewise identifies isolation by distance would add further weight to the idea that gene flow is responsible for the spread of resistance. If, as in *A. myosuroides*, a mosaic pattern of resistance is identified such that highly resistant populations are located in close proximity to susceptible populations (i.e., no evidence of isolation by distance and variation in resistance across populations), then it is inferred that populations are independently evolving resistance across the landscape. In this case, a pattern of high neutral genetic structure across populations would suggest rather limited gene exchange among populations, supporting the possibility that populations are independent evolutionary units. Hence, pairing an assessment of the level of resistance across the landscape with investigation of the genetic structure of a weed can allow us to identify the evolutionary units of herbicide resistance (Menchari et al. 2007)—and, likewise, provide initial information regarding the repeatability of the evolutionary process (Ralph and Coop 2010). Such examinations may also give insight into the ability of populations to respond to other abiotic and biotic selective agents following extreme bouts of selection (e.g., the likelihood of evolutionary rescue; Gonzalez et al. 2013). Finally, once the level of resistance and patterns of neutral genetic differentiation are characterized across the landscape, the more challenging question of how resistance has arisen—*that is,* through selection on standing genetic variation, or due to novel mutations across populations—can be addressed.

*Ipomoea purpurea*, the common morning glory, is a competitive crop weed within the Southeastern and Midwestern regions of the USA (Defelice 2001). This species has become an increasingly problematic species as the increased use of glyphosate (Culpepper 2006; Webster and Nichols 2012), which is the main ingredient in the widely used herbicide RoundUp. Previous work has uncovered both additive genetic variations in resistance to glyphosate and positive selection on resistance showing that the criteria for the evolution of a higher level of resistance are met (Baucom and Mauricio 2008). Additionally, historically preserved accessions exhibit genetic variability in herbicide defense, suggesting that the ability to evolve resistance in this species was present ancestrally (Baucom and Mauricio 2010). Although this species is considered an emerging glyphosate-resistant weed (reviewed by Sandermann 2006), we currently do not know whether the level resistance varies among populations across the species' range, nor do we know the extent to which populations may be connected *via* gene flow across the landscape. Here, and as part of our broader goal to determine whether resistance in this species has arisen from independent, novel mutations within separate populations, different regimes of selection across farms, or has spread *via* gene flow from a single or few sources, we examine both the level of herbicide resistance and the structure of neutral genetic variation across many natural populations of its range across North America. We performed a replicated glyphosate dose–response experiment and assessed the pattern of neutral genetic variation within this species using microsatellite markers to address the following specific questions: (i) Is there a geographic mosaic pattern of glyphosate resistance in *I. purpurea*, indicating that resistance has evolved independently in separate populations across the landscape, or is there a pattern of isolation by distance suggesting a single origin? (ii) Does the pattern of neutral genetic structure across this species' range provide evidence that populations are genetically isolated or that gene flow, whether historical or contemporary, has occurred or is occurring? and (iii) Is there evidence for migration between populations of the southeastern USA after glyphosate was put into widespread use across the landscape, indicating that contemporary gene flow is responsible for the spread of resistance? While the majority of studies that assess neutral genetic variation in herbicide-resistant weeds have investigated either predominantly outcrossing, wind-pollinated species, or alternatively highly selfing species, the work presented herein considers a species that exhibits a mixed-mating system, and one that, by all indications, is in the early stages of glyphosate-resistant evolution across the landscape.

## Materials and methods

### Field collections and greenhouse resistance screens

**Figure 1:**
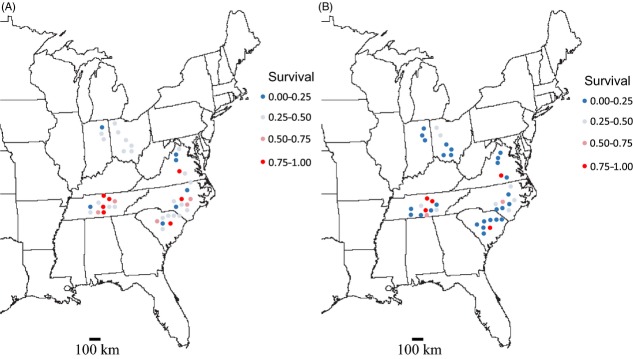
Survival 3 weeks post-RoundUp herbicide application among *I. purpurea* populations at the rate of (A) 1.7 kg a.i./ha and (B) 3.4 kg a.i/ha. The proportion survival within each population is indicated by color.

We collected leaf material and seeds from 44 populations of *I. purpurea* located within soya, cotton, corn or alfalfa fields selected at random from six states across the Midwestern and Southeastern USA (IN, OH, VA, NC, SC, TN; Fig. 1A, Table S1). We collected between 20 and 40 seeds and leaf material from a single maternal plant every 2 meters at each of our 44 sites until we had sampled from at least 30 individuals per population. Each population was sampled from a discrete agricultural field, which we assume to represent discrete units of selection. Populations were at least 5 km apart.

We planted two experiments to assay herbicide resistance across populations on June 11, 2013, in separate greenhouses at the University of Georgia Plant Biology Greenhouses (Athens, GA). Ten seeds from each population (one seed per maternal line) were scarified and then planted in pine bark soil in SC10 super containers (Stuewe and Sons, Tangent, OR) in each of six experimental treatments, described below. Individual plants were randomly assigned to racks that were then randomly assigned to flow trays (four racks per flow tray). Pots were watered daily, and flow trays were filled with water to prevent desiccation. Only plants that germinated prior to June 26, 2013, were included in the experiment—however, germination was high (88% overall) in both experiments and ranged from 50 to 100% among populations. A total of 4614 plants were used in our resistance assays, with 1995 and 2619 individuals planted in each experiment. The number of individuals per population and treatment combination used in the experiment can be found in Tables S1 and S2, respectively.

We measured the height of the stem and number of leaves of each individual 3 weeks after planting, when the majority of individuals were at the 5-leaf stage. Average height of the plants prior to herbicide application was 13.1 ± 0.3 cm. Plants were then sprayed with RoundUp PowerMax (Monsanto, St Louis, MO) at rates around the recommended field rate (1.54 kg ai/ha) of 0, 0.21, 0.42, 0.84, 1.70, and 3.40 kg a.i./ha (the 0 kg a.i./ha treatment was used as a water control) using a handheld, CO_2_-pressurized sprayer (Spraying Systems Co., Wheaton, IL). The same applicator treated each of the two replicate experiments. We sprayed plants at a rate of 187 liters/ha at 30 psi with a stride pace of 90 paces per minute at 1.5 meters above the plants. Three weeks after glyphosate application, we scored survival and the height of the living stem of each plant.

### DNA extraction and genotyping

We isolated DNA from approximately 18 maternal lines per population using a modified CTAB protocol developed by T. Culley (pers. comm.). All DNA samples were quantified by spectrophotometry and diluted to 20 ng/*μ*l for subsequent PCR. We identified 15 SSR loci that showed compatibility for multiplexing and were easily scorable of 20 that were previously described for *I. purpurea* (Table S3; Molecular Ecology Resources Primer Development Consortium et al. 2012). Forward primers were fluorescently tagged with 6-FAM, VIC, NED, or PET. All unlabeled and 6-FAM primers were obtained from Integrated DNA Technologies (Corralville, IA). The VIC, NED, and PET primers were purchased from Life Technologies (Carlsbad, CA).

Because fragment size and dye incompatibilities precluded running all 15 loci in one multiplex, we split loci into two multiplex PCRs. One multiplex consisted of 0.15 *μ*m of IP8, IP2, IP27, 0.20 *μ*m of IP31, and 0.25 *μ*m of IP34, IP18, and IP1. The second multiplex consisted of 0.05 *μ*m IP36, 0.10 *μ*m of IP47, 0.15 *μ*m of IP6, and 0.25 *μ*m of IP12, IP21, IP45, IP26, and IP42. Ten microlitre PCRs were run with Qiagen Master Mix (Valencia, CA). Thermocycler conditions consisted of 95°C for 3 min, 35 cycles of 94°C for 30 s, 50°C for 90 s, 72°C for 60 s, and a final extension at 72°C for 10 min on an Eppendorf (New York, New York) MasterCycler Pro thermacycler. One microliter of PCR product was used for fragment detection using an Applied Biosystems 3730 DNA Analyzer (Carlsbad, CA) at the Cornell Life Sciences Core Facility (Ithaca, NY). An ABI GS500 for multiplex 1 and GS600 for multiplex 2 size standards were used for fragment length comparison. All sample genotypes were analyzed using Applied Biosystems PeakScanner 1.0 analytical software (Carlsbad, CA). A PP (Primer Peaks adjustment) sizing default was used for the analysis.

### Data analysis

We included populations with at least 10 germinants per experiment (all treatments combined) in the analyses of spatial variation of herbicide resistance. We first report a species dose–response curve and then follow with patterns of survival across populations at doses above the recommended field rate.

### Dose–response curve

Preliminary analysis showed a significant effect of replicate experiment on the proportion that died. Thus, we used residual values after accounting for the effect of greenhouse experiment to estimate our dose–response curves. The use of residual is recommended by Kelly and Rice (1990) to smooth curves in dose–response analyses and has been performed in other dose–response contexts (Kilsby et al. 2000). Replicate experiments differed primarily due to a higher rate of death in one greenhouse at both the 1.7 and 3.4 kg a.i./ha herbicide levels, but the correlation between proportion survival per population across each greenhouse was moderate and significantly different from zero across all concentrations (*R* = 0.480, *P* = 0.004), showing that although we identify differences in the survival of plants in separate greenhouse experiments, the populations performed similarly relative to one another in the different experiments. To estimate the dose–response curve, we first fit the residuals by regressing a general linear model of survivorship on experiment with a binomial distribution to account for the effect of replicate. The residual data were then fit to a Weibull 2.4 parameter model (Weibull 1951) using the drc package (Ritz and Streibig 2013) in R (R Development Core Team, 2012). The Weibull 2 model was used to extrapolate the effective dose at eliminating 50% of a population (ED_50_) was expressed as:


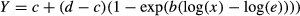


where *Y* is the response (survivorship), *c* is the upper limit of the curve, *d* is the lower limit of the curve, *e* is the ED_50_, and b is the relative slope around *e*. We first estimate a species-level ED_50_ using all individuals, followed by a regional (Southeastern and Midwestern USA) ED_50_ value, and ED_50_ values per state. We included state in the models because we hypothesized that management policies and herbicide procedures might vary at the state level, and this could influence the level of resistance among states.

### Survival following herbicide application

We assessed survival at the 1.7 and 3.4 kg a.i./ha treatment levels (a rate that is similar to the suggested field rate of 1.54 kg a.i./ha and a dose that is twice that) to determine whether there was a significant effect of population, state, and/or region of origin on survival postherbicide. To do this, survival was modeled as a binary character (1/0) in a generalized linear model with a binomial distribution using the lmer option of the lme4 package (Bates 2010) in R (R Development Core Team, 2012). Fixed effects in the model were herbicide treatment, replicate experiment, and state while random variables included population nested within state and the interaction between population and herbicide treatment. We estimated a 95% confidence interval for the species survival by bootstrapping across populations using a nonparametric bootstrapping method in the boot package (Canty and Ripley 2014) in R (R Development Core Team, 2012).

### Spatial autocorrelation

We calculated Moran's I to determine whether there was a correlation between survival following herbicide application and geographic distance. Specifically, we calculated the correlation between survivorship and its spatial lag using the Spatial Autocorrelation Analysis in Macroecology (SAM 4.0, Rangel et al. 2010). The significance of the I value was determined by permuting around 0, where a value of 0 would reflect no spatial pattern (or spatial dispersion) in the data (Bivand et al. 2008). We further performed a principle coordinates analysis (PCoA) on the absolute value of the pairwise difference in survival (ΔResistance = abs(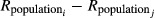) using the covariance-standardized option in GenAlEx (Peakall and Smouse 2006) across populations to determine whether populations clustered according to geographic origin or to resistance status (i.e., survival).

### SSR error rate, reliability, independence, and neutrality

We evaluated SSR loci for reliability, independence, and neutrality under mutation–drift equilibrium to make sure interpretations of downstream analyses were appropriate (i.e., assumptions made by analyses were not grossly violated). We used MicroChecker (Van Oosterhout et al. 2004) to check for scoring errors per population, which could result from stuttering, large allele dropouts, and null alleles (Dewoody et al. 2006). Each SSR locus was tested for Hardy–Weinberg equilibrium (HWE) per population using the Hardy–Weinberg exact test in Genepop on the Web (Suboption 3: Probability test; Raymond and Rousset 1995; Rousset 2008). SSR loci were also tested for linkage disequilibrium for each pair of loci in each population using the genotypic linkage disequilibrium test with default Markov chain parameters in Genepop. A global test of LD for each pair of loci was performed across populations using Fisher's method. We then applied a sequential Bonferroni correction (Miller 1981) to correct for multiple tests. Loci were independently scored twice to check for accuracy, and we recorded the scoring error within the species for 200 samples. Loci that did not amplify after two independent attempts were scored as missing data; the frequency of missing data for each locus is reported in Table S3.

### Genetic differentiation

We assessed patterns of neutral genetic variation across this species' range using a variety of metrics to determine whether genetic diversity and population structure were influenced by recent selection *via* herbicide application and, in addition, to assess the likelihood that gene flow could introduce, or has historically introduced, resistance alleles to once-susceptible populations. We first determined the genetic structure of this species by estimating Weir and Cockerham's (1984) θ using SPAGeDi-1.2 (Hardy and Vekemans 2002). A confidence interval around the global *F*-statistic was estimated with 1000 permutation per locus and 1000 jackknifed replicates to detect significant deviation from 0. This confidence interval was used to compare with previous estimates of differentiation within the species (Epperson and Clegg 1987). We evaluated multilocus estimates of *R*_ST_, a measure of genetic differentiation using a stepwise mutation model of marker evolution. Pending no significant difference between *R*_ST_ and *F*_ST_ estimates, we report only differentiation using those based on *F*_ST_. In preliminary analyses, we assessed the influence of null alleles on our measure of genetic differentiation using FreeNA (Chapuis and Estoup 2007) by excluding null alleles and comparing *F*_ST_ to nonadjusted estimates. As the two estimates were comparable, we report a nonadjusted *F*_ST_ (95% C.I. *F*_STadj_: 0.071–0.171, 95% C.I. *F*_STunadj_: 0.071–0.168).

We next implemented a hierarchical amova to test the level of genetic differentiation across regions (MW, SE), states within regions (IN, OH, VA, SC, NC, TN), and populations nested within states using GenAlEx v. 6.1 (Peakall and Smouse 2006). To assess the potential for contemporary or historical admixture between populations, we clustered individuals across the Southeastern and Midwestern USA using STRUCTURE v. 2.2.3 (Pritchard et al. 2000) with an admixture model and an MCMC length of 400 000 iterations (100 000 burn-in). Likelihood values of the number of clusters, Ln(*P*(*D*)), were assessed from five runs using a range of k values from 1 through 35. We used the delta-k method (Evanno et al. 2005) to determine the most likely number of clusters in our data set. We also performed a principal coordinates analysis to determine whether the neutral genetic variation of populations clustered together according to geographical distance in GenAlEx v. 6.1 (Peakall and Smouse 2006), and further, tested for isolation by distance using Rousset's (1997) linearized *F*_ST_, *F*_ST_/(1−*F*_ST_), and Cavalli-Sforza distance over the natural log of geographic distance using ISOLDE, Option 6 in GenePop (Raymond and Rousset 1995; Rousset 2008).

We then performed pairwise comparisons of genetic structure between populations to determine whether populations were significantly differentiated from one another and thus less likely to share a similar evolutionary history. Pairwise estimates of genetic differentiation among all sampled populations were estimated by *F*_ST_ (Weir and Cockerham 1984) using the program FSTAT v. 2.9.3 (Goudet 2001), and their significance was determined using 1000 permutations and a sequential Bonferroni correction for multiple tests. Finally, we estimated Nei's pairwise genetic distance and a subsequent principle coordinates analysis using the covariance-standardized option in GenAlEx v. 6.1 (Peakall and Smouse 2006).

### Approximate Bayesian computation (ABC) analysis

**Figure 2:**
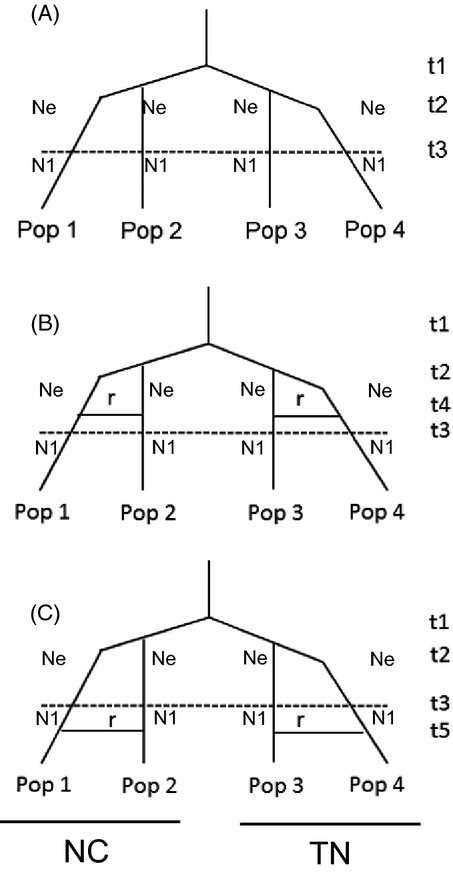
Three simple scenarios used in the ABC analyses of *Ipomoea purpurea* populations sampled from NC and TN. Four populations each trial were used to model (A) no gene flow among populations, (B) admixture (denoted *r*) that occurred before the widespread use of the herbicide, and (C) admixture after widespread glyphosate use. The origin of the populations occurred at *t*1, or the time at which the species was identified as an agricultural weed in the United States, taken from (Defelice 2001). Population divergence between TN and NC populations is indicated by *t*2. Widespread glyphosate use is indicated in *t*3 and denoted with a dotted line, corresponding to the year (1974) at which glyphosate was released for commercial use in US agriculture. Admixture events are indicated either with *t*4 (gene flow prior to glyphosate use) or *t*5 (gene flow after glyphosate use). Also included in the model were the effective population size of each population at *t*2 (*N*_e_) and subsequent effective size postglyphosate bottleneck (*N*_1_). Parameter estimates are shown in Table S4.

We next used a Bayesian coalescent approach (approximate Bayesian computation; Beaumont 2010) to examine the likelihood that migration occurred recently between resistant populations in the southeastern USA (see Results) compared to a scenario of gene flow between populations prior to the widespread use of glyphosate across the landscape. If the former scenario were more likely, we would reason that contemporary gene flow (such as through the movement of contaminating morning glory seed between farms in seed lots) is likely to be responsible for resistance across populations. If, however, the latter scenario of migration before the widespread use of glyphosate were the more likely one, we would infer that resistance has independently evolved in separate populations. Although ABC analysis can be used to make inferences about complex population histories, estimate population parameters such as effective population size (Tallmon et al. 2008), and has recently been used to model many different scenarios of herbicide resistance evolution (Okada et al. 2013), we elected to model the relatively simple alternative scenarios of migration between *I. purpurea* populations pre- or postglyphosate use. We employed the software DIYABC v 2 (Cornuet et al. 2008) to test three scenarios (Fig. 2) using the microsatellite data from North Carolina and Tennessee populations—two areas of the landscape where we observed the highest resistance (see Results). The first scenario assumed no admixture across populations. The second scenario assumed admixture before the use of glyphosate (at time *t*4) and a third scenario assumed admixture after the use of glyphosate (time *t*5) within NC and TN regions.

We estimated *t*5 as the number of generations that have occurred since 1974 (a range of 2–38), which is the year that RoundUp was approved for chemical weed control (Duke and Powles 2008). We included a bottleneck event across all populations around the start of glyphosate use in 1974, where population size reduced by 90%, a target value of many herbicides (denoted by dashed line at time *t*3), from an initial effective population (*N*_e_) size ranging between 250 and 1000 individuals, which encompasses the range of previously estimated *N*_e_ for *I. purpurea* (Gonzales et al. 2012). Additional time points at which weedy populations were first observed in the region and diverged (*t*1 and *t*2, 180-210 generations from present) had previously been described in Defelice (2001) and were included in the models. Parameter values for tested models can be found in Table S4. The probability estimates of model scenarios were compared using posterior probabilities from a local logistic regression of the scenario set, and 100 000 runs were assessed per scenario. We ran these simulations using 15 populations over four trials with four populations per trial. Each trial included two randomly chosen populations from TN and NC each with one population (30) used twice across trials.

## Results

### Dose–response

**Figure 3:**
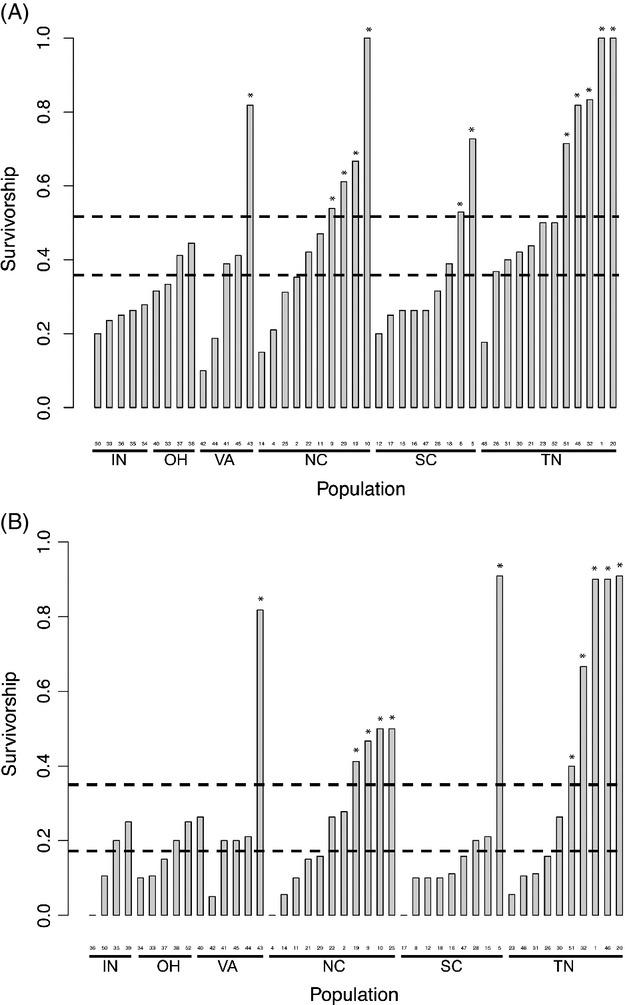
*Ipomoea purpurea* populations greater or less than the species' average of resistance at (A) 1.7 and (B) 3.4 kg a.i./ha. Horizontal dashed bars indicate the bootstrap estimates of the 95% confidence interval around the species' mean. Asterisks indicate populations that fall outside the 95% confidence interval.

The overall species-level ED_50_ estimate for *I. purpurea*, based on survival, was 1.6 kg a.i./ha (95% CI: 1.12–2.10), which is similar to the manufacturer's recommended field dose of 1.54 kg a.i./ha. Twelve populations, all of which were from VA, SC, NC, and TN, exhibited a proportion survival that was significantly higher than the species average, *that is,* resistance values that were greater than the species 95% CI (Figs 1 and 3A). Nineteen populations fell significantly below the species average—12 of these were from the Southeastern USA (SC, NC, and TN) and seven were from the Midwestern USA (IN and OH) (Fig. 3A). Population-level ED_50_ estimates were considerably variable; however, overall, populations within the Southeastern USA, principally North Carolina and Tennessee, exhibited ED_50_ values above the species-level 95% CI, whereas populations in the Midwestern USA, mainly Ohio and Indiana, exhibited response levels below the average (Table S5) although the difference was not statistically significant.

### Spatial variation in resistance

**Figure 4:**
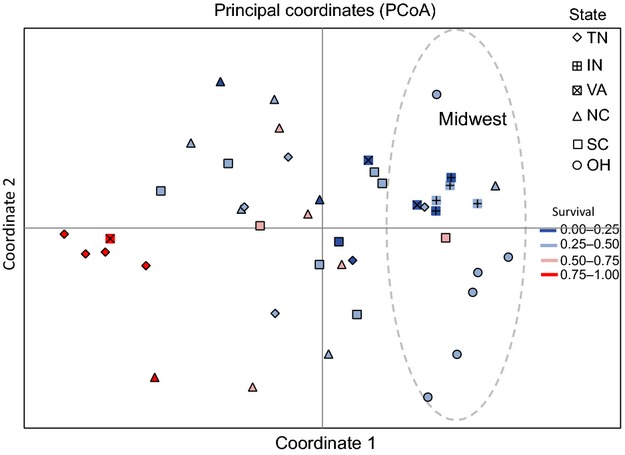
PCoA of pairwise differences in resistance values between populations at 1.7 kg a.i./ha. Populations are assigned to state (shape) and resistance level by color (red-blue gradient). Coordinate 1 explained 16.4%, and coordinate 2 explained 13.5% of the variation in survival. The dashed open circle represents the coordinate space representing all of the Midwestern US populations.

Although the species' ED_50_ value was very close to the recommended field dose, we found a significant effect of population of origin (*χ*^2^ = 145.34, *P* < 0.001) and state on survival (*F*_5,8182_ = 2.540; *P* = 0.030, Table 1), indicating the presence of geographic variation in the level of resistance. We found no effect of region, although we observed that northern sites tended to exhibit lower survival than southern populations at 1.7 and 3.4 kg a.i./ha (Figs 1A,B and 3A,B, respectively). The interaction between population and herbicide dose (Population × Treatment *χ*^2^ = 0.040; *P* = 0.980, Table 1) was not significant, suggesting that even though some populations exhibited higher survival then others, the populations responded in a relatively consistent manner to the different herbicide doses, namely increasing death at a higher herbicide application rate. There was significant spatial autocorrelation of resistance at distances at a local scale (within 40 miles, Moran's *I* = 0.829, *P* = 0.013), but we observed no isolation by geographic distance across all sampled populations in survival (*R* = 0.020, *P* = 0.269). The Southeastern USA exhibits greater diversity in the proportion survival per population than the Midwestern USA; although the most resistant populations do not, in general, cluster together in a PCoA (Fig. 4). An exception to this was the highly resistant TN populations, which tended to cluster together.

**Table 1:** Generalized linear mixed-effects model of plant survival as a response of the fixed effects of treatment, experimental replicate, state, and random effects of populations (nested within state) and the population × treatment interaction. Shown are the degrees of freedom (df), *F* or *χ*^2^ statistic, and associated *P* value

Effect	df	*F*	*P*
Fixed Effects			
Treatment	5	155.47	<0.001
Replicate	1	8.28	0.004
State	5	2.54	0.026
Random Effects		*χ*^2^	*P*
Population (State)	2	145.34	<0.001
Population (State) × Treatment	2	0.04	0.980

Populations used for this test are listed under the column ‘2012 Survivorship’ in Table S1.

### Genetic diversity and differentiation

Information on scoring errors, deviations from Hardy–Weinberg Equilibrium and locus pair linkage disequilibrium can be found in the Supporting information section. The number of alleles per locus × population ranged from 1.60 to 2.27 (mean = 2.00), and allelic richness per multilocus genotype × population combination ranged between 1.23 and 1.37 (mean = 1.30). Expected (*H*_e_) and observed (*H*_o_) heterozygosity ranged between 0.230–0.372 (mean = 0.304) and 0.191–0.400 (mean = 0.294), respectively (Table S6).

The amova uncovered evidence for low but significant genetic differentiation across region (*F*_RT_ = 0.043, *P* = 0.001, Table 2), states within regions (*F*_SR_ = 0.119, *P* = 0.001), and populations within states (*F*_PS_ = 0.157, *P* = 0.001). The majority of genetic variation in *I. purpurea* is found within populations (*F*_IT_ = 0.428, *P* = 0.001).

**Table 2:** Analysis of molecular variance (amova) of neutral genetic data. Shown are the main effects of Region (Midwestern and Southeastern USA), State, Population and Individual, *F*-statistic, and *F* and *P* values

Effect	*F*-statistic	*F*	*P*
Region	*F*_RT_	0.043	0.001
State (Region)	*F*_SR_	0.119	0.001
Population (State)	*F*_PS_	0.157	0.001
Individual	*F*_IT_	0.428	0.001

We estimated Weir and Cockerham's (1984) *F*_ST_ across the species' range to be 0.127, (95% CI: 0.071–0.183), which is lower than a previous estimate using floral color (*F*_ST_ = 0.218, Epperson and Clegg 1987). We detected no difference between *R*_ST_ and *F*_ST_ estimates (*R*_ST_ = 0.068, 95% CI: 0.0681–0.122). One hundred and eight (21%) of 595 pairwise-*F*_ST_ values between populations were significantly greater than 0, and ranged from 0.035 (Burgaw, NC and IN10; Table S7) to 0.274 (Hare Road, NC- Willis Grove, TN; Table S7). We found no evidence of genetic differentiation among 79% of populations; the majority of significant *F*_ST_ values were between populations sampled from different states (86%). Of the significant *F*_ST_ values among states, the majority were observed between populations in TN and NC (15%) and SC (15%)—interestingly, these were states in which we observed highest levels of resistance. There was, however, no indication that resistant populations exhibited more or less differentiation compared to other populations, as the majority (64%) of the significant pairwise-*F*_ST_s were among populations that exhibited resistance values within the species' 95% CI, and, less than 2% of the significant pairwise-*F*_ST_s were between resistant and susceptible populations.

**Figure 5:**
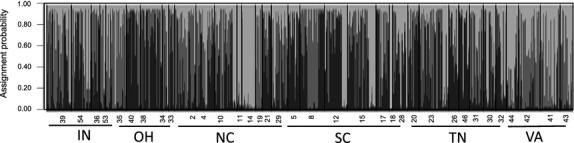
STRUCTURE assignment of individuals to genetic clusters. Small bars represent the assignment of individuals to clusters, with sampling locations differentiated by thick black lines for 35 populations sampled. Shown are each population denoted by State: IN = Indiana, OH = Ohio, NC = North Carolina, SC = South Carolina, TN = Tennessee, VA = Virginia, and population ID number.

Further, although we found a moderate level of genetic differentiation across populations sampled from North America, our STRUCTURE analysis uncovered a pattern of widespread migration and admixture among individuals within populations (Fig. 5). The most likely number of genetic clusters within the sampled range for *I. purpurea* was *k* = 3 (ln(*P*(*D*) = −8265.7). All 3 genotypic clusters were found within individuals sampled from North Carolina suggesting that populations within this state are either the source of introduction for other weedy populations or this state has had multiple introductions of different seed lots.

**Figure 6:**
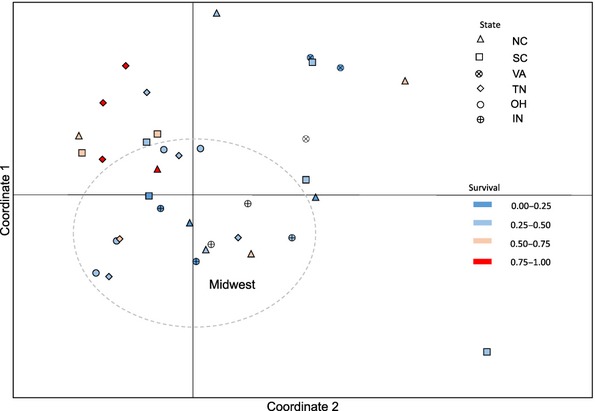
Principle coordinate analysis of pairwise Nei's genetic distance. Populations are assigned to state (shape) and resistance level by color (red-blue gradient). The proportion of genetic variance explained by coordinates 1 and 2 were 8.9 and 6.3%, respectively. The dashed open circle represents the coordinate space representing all of the Midwestern US populations.

Our PCoA of neutral genetic variation revealed a slight clustering of Midwestern US populations, which had similarly been found in the amova result for regional genetic differentiation (*F*_RT_ = 0.043, *P* = 0.001). However, these populations were contained within the range of variation across the Southeastern US populations (Fig. 6), and the first two axes of the PCoA explained only 8.9% and 6.3% of the variation. Thus, geography explains only a small portion of the neutral genetic diversity of this species, and the majority of neutral genetic variation across this species' range in the USA is present within the southern populations. Wilcoxon tests on the first 2 axes of the principle coordinates found no difference, across either axis, for the populations when assigned either ‘resistant’ (<50% death, *N* = 11) or ‘susceptible’ (>50% death, *N* = 22) in PC1 or PC2 mean scores (Axis 1: W = 157, *P* = 0.175; Axis 2: W = 106, *P* = 0.585). Hence, there was no indication that the neutral genetic variation of this species clustered according to resistance status rather than geography, as would be expected if propagules from, for example, resistant TN populations had migrated to the resistant Carolina populations and established and/or admixed. We did not uncover evidence of isolation by distance using linearized *F*_ST_ over geographic distance (*R*^2^ = 0.012, *P* = 0.142), nor did we uncover significant isolation by distance measured as the Cavalli-Sforza Edwards chord distance (*R*^2^ = 0.010, *P* = 0.192). Pairwise estimates of Nei's genetic distance similarly did not correlate with geographical distance (*R* = −0.065, *P* = 0.11), reinforcing our finding of either widespread gene flow across populations or colonization following a recent bottleneck.

### Approximate Bayesian computation analysis

We found overwhelming support for admixture prior to glyphosate use (Table 3, average posterior probability across four trials = 0.9515, 0.9413–0.9617) rather than the scenario of gene flow and admixture after 1974, or the time that glyphosate was put into widespread use (average posterior probability = 0.0474, 0.0372–0.0575). This scenario was also more likely than the scenario of no admixture (average posterior probability = 0.0012, 0.0004–0.0021).

**Table 3:** The posterior probabilities and associated confidence intervals for different histories of *Ipomoea purpurea* populations, based on the logistic estimate from the ABC analysis. Logistic regressions were performed using three scenarios: Scenario 1, no admixture; Scenario 2, admixture before the widespread use of the herbicide; and Scenario 3, admixture after the herbicide was put into widespread use in agriculture. The populations used in each trial are shown, along with the posterior probability and associated 95% confidence interval of each scenario for 4 replicate trials and their overall average. Posterior probabilities that are significant are indicated in bold text

		Scenario 1		Scenario 2		Scenario 3	
Trial	Populations	Posterior Probability	95% Confidence Interval	Posterior Probability	95% Confidence Interval	Posterior Probability	95% Confidence Interval
1	2,14,30,31	0.0001	0.0000–0.0002	**0.9918**	0.9889–0.9948	0.0081	0.0051–0.0110
2	29,4,20,26	0.0005	0.0001–0.0009	**0.9682**	0.9596–0.9768	0.0313	0.0227–0.0398
3	11,10,46,23	0.0001	0.0000–0.0011	**0.9399**	0.9244–0.9554	0.0600	0.0445–0.0755
4	19,21,30,32	0.0039	0.0015–0.0063	**0.9060**	0.8923–0.9196	0.0901	0.0766–0.1036
Average		0.0012	0.0004–0.0021	**0.9515**	0.9413–0.9617	0.0474	0.0372–0.0575

## Discussion

Our comprehensive analysis of herbicide resistance and neutral genetic variation in the weed *Ipomoea purpurea* has uncovered four major findings. First, while we find that the overall species ED_50_ value is similar to the recommended field dose, we observed considerable spatial heterogeneity in resistance with some populations exhibiting ∼100% survival at high doses of glyphosate and others exhibiting high susceptibility. Second, we found little indication that the level of resistance exhibits isolation by distance suggesting that resistance across populations of this species results from either novel mutations within each population or is a result of differing rates and exposures to herbicide application across the landscape. Strikingly, we uncovered little evidence for a genetic signal via isolation by distance or strong geographic structuring in our assay of neutral genetic variation—we instead detected a pattern of widespread migration and admixture across this species' range in the USA. Finally, our ABC analysis indicated that gene flow between populations most likely occurred prior to the widespread use of the herbicide rather than very recently. Overall, these results support the idea that some populations of *I. purpurea* have rapidly developed higher levels of resistance to this herbicide within a short time frame (as the widespread use of RoundUp beginning in the early 1990's) and that it is unlikely increased resistance is due to contemporary gene flow between populations, but rather, results from independent regimes of selection *via* the herbicide. We discuss each of these main points below.

### The geographic mosaic of herbicide resistance

We uncovered broad variation in resistance across populations collected from the Southeastern and Midwestern USA, with a pattern that indicates herbicide resistance is evolving independently in a mosaic of hotspots. Although we found that the species average level of resistance is comparable to the suggested field dose (1.54 kg a.i./ha), we uncovered populations that exhibited very high or very low survivorship postherbicide application. Populations that exhibited high survival and thus high resistance did not appear to cluster in one region of the landscape—*that is*, resistant populations were located near susceptible populations—suggesting that resistance has independently evolved across disparate areas of this species' distribution. This pattern of potentially independent resistance hotspots has been shown in other resistant weed species (Menchari et al. 2007; Delye et al. 2010), and can result from differences in management practices across geography (Delye et al. 2010), differences in the structure of genetic variation within populations across the landscape (Mopper et al. 2000; Brodie et al. 2002; Bernhardsson et al. 2013; Delye et al. 2013) or a combination of differences in herbicide use patterns and variation in the standing genetic variation of populations. That we detected no evidence for isolation by distance in the level of resistance further strengthens the case that resistance has evolved independently several times across populations of this species. We did, however, uncover evidence for local geographic structuring of resistance (within 40 miles). This finding, in addition to the lack of isolation by distance across all populations, suggests that the individual farm is the independent unit of resistance evolution, a conclusion that is similar to that of ACCase-resistant blackgrass in France (*Alopecurus myosuroides*) (Delye et al. 2010).

Although we uncovered evidence of a geographic mosaic of resistance, we also found regional differences in survivorship—states in the Southeastern USA tended to have higher ED_50_ estimates and survivorship compared to Midwestern US populations. Because management practices are often regulated at the state level, it is possible that the difference in resistance between regions may result from differences in the recommended dose across areas. For example, TN has the highest recommended application rate in corn (0.75–1.5 lb a.i./ha, Steckel et al. 2014), whereas OH and IN have the lowest recommended application rate (0.56–1.12 lb a.i./ha, Loux et al. 2013); our ED_50_ values for TN and OH and IN align with the upper end of these recommended rates (TN = 1.5 lbs a.i./ha; OH = 1.12 lbs a.i./ha; IN = 1.12 lbs a.i./ha) as does survival postherbicide (Fig. 1A).

### Patterns of genetic variation and population structure

With some notable exceptions (Okada et al. 2013), the neutral genetic variation of many weeds exhibits little structure or spatial patterning (e.g., Bommarco et al. 2010; Delye et al. 2010; Campitelli and Stinchcombe 2012), potentially due to either their recent expansion across the landscape, few barriers to gene flow, or human-mediated modes of dispersal (e.g., dispersal through farm machinery or through contaminated crop seed; Thill and Mallory-Smith 1997; Owen and Zelaya 2005). While we find evidence for low-to-moderate genetic structure across populations (*F*_ST_ = 0.127, *P* = 0.001), we find little evidence for a geographic pattern to that structure beyond the slight clustering of Midwestern US populations identified in the PCoA. In particular, we found no isolation by distance within the species, suggesting a scenario of either widespread gene flow between populations or their relatively recent colonization.

We hypothesize that recent colonization and introduction patterns are responsible for the lack of geographic structure in this species. *Ipomoea purpurea* is a very popular ornamental that has been re-introduced to the Southeastern USA (Defelice 2001; Fang et al. 2013) many times following flower color domestication (Glover et al. 1996), and this species does particularly well in warm climates as it is native to central Mexico. Thus, the Southeastern USA in particular may have experienced repeated re-introduction and establishment of this species, following which subsequent range expansion or colonization into more northern areas occurred. Perhaps the presence of *some* genetic structure and yet evidence for migration and admixture between populations of this species is due to the re-introduction of a limited but variable pool of germplasm, a scenario similar to that posited for *I. purpurea's* sister species, *I. hederacea* (Campitelli and Stinchcombe 2014). The Carolinas (specifically NC) have a relatively high density of populations of *I. purpurea* compared to other states—and populations within this range contain all of the genotypes that we detected in our survey, suggesting NC as a possible source for subsequent introductions into other areas of the Southeastern and the Midwestern USA.

The low level of genetic structure across populations of this species could also be due to contemporary migration and admixture between populations, and this scenario would strongly suggest that gene flow could be a major driver of resistance evolution in this species. If this were the case, however, we would expect to see patterns of isolation by distance in either our phenotypic resistance data or in patterns of neutral genetic variation—admittedly, a line of reasoning that assumes little chance of long-distance propagule or pollen movement *via* human influence. Thus, the overall mosaic pattern of phenotypic variation in this system suggests resistance has emerged through independent evolutionary events, whereas the genetic data provide little evidence that the populations are genetically independent. To resolve these two patterns, we used a Bayesian coalescent approach and explicitly considered two scenarios of population connectedness—one in which migration among populations occurred primarily before the commercial approval and widespread use of the herbicide (1974; Fig. 1 of Baucom and Mauricio 2004) and another that examined the probability associated with very recent, postwidespread glyphosate use. This analysis consistently identified support for the preherbicide migration scenario compared to a scenario of recent, and postherbicide use migration, pointing to the independent evolution of resistance across populations in a mosaic fashion.

An interesting and remaining question is whether or not the potential independent evolution of resistance is due to selection on pre-existing and hence similar genetic variation, or due to novel mutations in the same or different genomic architecture. Previous work in this species has identified genetic variation for glyphosate defense in accessions of *I. purpurea* collected in the 1980's, prior to the widespread use of RoundUp in the early 1990's (Baucom and Mauricio 2010), such that the genetic potential for resistance was present ancestrally within this species. This would suggest that independent and increasing regimes of selection on standing genetic variation *via* the use of RoundUp are responsible for resistance uncovered in separate populations. Our data taken in a geographic context also show that it is highly unlikely that there was a single origin of resistance, as the landscape of resistance is heterogeneous even at small areas. It is more plausible that rapid and relatively recent (post-Columbian), but still historical gene flow is responsible for the low genetic differentiation. Populations then went through rapid adaptation of increased resistance across separate areas within the past ∼20 years due to the prevalent use of Roundup herbicide. To conclusively rule out the possibility that rare gene flow events may have introduced resistance alleles across disparate areas, however, we will need to determine whether the genetic basis of resistance across populations differs, and perform an analysis of the phylogeographic history of resistance alleles.

## Data archiving statement

Data deposited in the Dryad Digital Repository: http://dx.doi.org/10.5061/dryad.p3v3s.

## Supporting Information

**Appendix S1.** Results. **Table S1.** Site and location information for *I*. *purpurea* populations used in both the resistance assay as well as the genetic differentiation and diversity study. **Table S2.** Number of individuals used for each population and treatment combination. **Table S3.** Description of Simple Sequence Repeat loci developed in Kuester et al. (2012). **Table S4.** Parameters used in the DIYABC analysis. **Table S5.** The effective dose across *I. purpurea's* range. **Table S6.** Descriptive statistics per site across multilocus genotypes. **Table S7.** Analysis of Molecular Variance (amova) of neutral genetic data for which we had at least 18 individuals per population (15 populations, bolded in Table S1). **Table S8.** Description of Simple Sequence Repeat loci developed in Kuester et al. (2012). **Table S9.** Likelihood values (Ln(*P*(*D*)) from STRUCTURE analysis with only populations where sample size was greater than 18 individuals per population. **Figure S1**. STRUCTURE assignment of individuals to two genetic clusters. Small bars represent the assignment of individuals to clusters, with sampling locations differentiated by thick black lines for 15 populations sampled with sample sizes greater than 18 individuals.
