# Supplementary material for: The geographic mosaic of herbicide resistance evolution in the common morning glory, Ipomoea purpurea: Evidence for resistance hotspots and low genetic differentiation across the landscape
Source: Evol Appl. 2015 Aug 13;8(8):821–33. doi: 10.1111/eva.12290 (PMC4561571; doi:10.1111/eva.12290)
Supplement: Supplementary file 1 [file eva0008-0821-sd1.docx]

## Supporting Information

Article title: The geographic mosaic of herbicide resistance evolution in the common morning glory, *Ipomoea purpurea*: Evidence for resistance hotspots and low genetic differentiation across the landscape

The following Supporting Information is available for this article:

**Results**

*Scoring errors*—Scoring errors were assessed in locus x population combinations using MicroChecker (Van Oosterhout et al. 2004). We detected potential scoring errors due to stutter—an error resulting from slippage of the polymerase during PCR—in 6 of 15 loci (2-20% across population, mean = 3.8%). Null alleles were detected in 10 of 15 loci (2-20% of populations, mean = 8.0%). Large allele dropout—under-amplification of larger allelic variants—was not detected in our dataset. We found no significant difference in genetic structure (F_ST_ = 0.113, P = 0.01) after removing 3 loci where stutter was found in excess of 10%. F_ST_ estimates adjusted for the presence of potential null alleles were not significantly different from those of unadjusted estimates.

*Hardy-Weinberg Equilibrium*— Each SSR locus was tested for Hardy-Weinberg Equilibrium (HWE) per population using the Hardy Weinberg Exact Test in Genepop on the Web (Sub-option 3: Probability test; Raymond and Rousset 1995; Rousset 2008). Significant P-values were estimated using Fisher’s exact test. Of 525 loci x population combinations, we detected 161 (30%) that deviated from Hardy Weinberg equilibrium. We detected only slightly elevated genetic structure (F_ST_ = 0.153, 95% CI: 0.082-0.229) after removing loci that were not in HWE (>30 % occurrence of significant deviations from HWE across populations), compared to the non-adjusted F_ST_.

*Linkage Disequilibrium—* SSR loci were also tested for linkage disequilibrium for each pair of loci in each population using the genotypic linkage disequilibrium test with default Markov chain parameters in Genepop. A global test of LD for each pair of loci was performed across populations using Fisher’s method. We then applied a sequential Bonferroni correction (Miller 1981) to correct for multiple tests. We found no evidence for linkage disequilibrium across each locus pair x population combinations.

*Scoring accuracy*—Of 200 multilocus genotypes checked for accuracy of scoring by double scoring them, we detected the following manual-scoring error rates for each of the loci: IP1 (3.1%), IP2 (3.4%), IP6 (1.7%), IP6 (1.7%), IP8 (1.5%), IP12 (1.5%), IP18 (2.0%), IP21 (2.0%), IP26 (1.7%), IP27 (4.7%), IP31 (2.1%), IP34 (2.3%), IP36 (5.5%), IP42 (1.8%), IP45 (1.6%) and IP47 (2.1%).

We detected the following amounts of missing data—defined here as the lack of a clear genotype at a locus after two attempts of PCR amplification and fragment size analysis—for each locus:

IP1 (25.17), IP2 (10.64%), IP6 (8.87%), IP8 (23.06%), IP12 (17.90%), IP18 (24.84%), IP21 (15.97%), IP26 (18.06%), IP27 (21.12%), IP31 (2.90%), IP34 (7.10%), IP36 (0.00%), IP42 (23.71%), IP45 (20.65%) and IP47 (14.35%). We interpret these results as indicating the frequency of homozygous null alleles in the dataset.

*Comparison of full SSR dataset to a subset of populations with sample sizes greater than 18 individuals—*When we reduced our dataset to only populations with at least 18 maternal lines (15 populations), we found that genetic differentiation between regions (0.043 vs 0.038) and among states (0.119 vs 0.090) and populations (0.157 vs 0.124) remained significantly different from zero, and that estimates were slightly reduced (Table 2 and Table S7). On the other hand, estimates of observed (0.29 vs 0.29) and expected (0.29 vs 0.30) heterozygosity remained virtually unchanged. In addition, estimates of F_ST_ fell well within 95% confidence limits (F_ST_ _full data_ = 0.125 + 0.022, F_ST_ _reduced data_ = 0.126 + 0.029, Tables S3 and S8). A STRUCTURE analysis revealed the most-likely number of groups to be 2 (L(P(D)) = -5294.4, Table S9) and again, no visible structure was observed across this partition (Figure S1) using the restricted data set compared to the full set of populations (*i.e.,* 18 vs 35 populations).

**Table S1.** Site and location information for *I*. *purpurea* populations used in both the resistance assay as well as the genetic differentiation and diversity study. Shown are the population number, state of each population, the field type from which seeds were sampled, and Latitude and Longitude GPS coordinates. Also shown are the number of individuals used in the dose response experiment and the number of individuals used for SSR genotyping. Populations marked as NA in this column were not used in the genetic differentiation study, and bolded numbers in this column indicate the populations used when subsampling the data to those populations with greater than 18 individuals.

| **Population Number** | **State** | **Field Type** | **Lat** | **Long** | **Number Inds for ED50** | **Number of Inds for SSR** |
| --- | --- | --- | --- | --- | --- | --- |
| 1 | TN | Corn | 35.775237 | -85.903419 | 62 | NA |
| 2 | NC | Corn | 34.595714 | -77.927484 | 101 | **24** |
| 4 | NC | Corn | 34.556672 | -79.125602 | 115 | **22** |
| 5 | SC | Soy | 33.859875 | -79.909072 | 69 | 14 |
| 8 | SC | Corn | 34.297195 | -79.991259 | 107 | **28** |
| 9 | NC | Soy | 34.924044 | -77.796171 | 87 | NA |
| 10 | NC | Soy | 34.983161 | -78.039309 | 68 | **26** |
| 11 | NC | Corn | 34.527135 | -78.756704 | 116 | 5 |
| 12 | SC | Cotton | 34.145812 | -79.865313 | 117 | **27** |
| 14 | NC | Soy | 35.424763 | -77.917121 | 116 | 16 |
| 15 | SC | Soy | 34.104209 | -79.073735 | 118 | **33** |
| 16 | SC | Alfalfa | 34.10535 | -79.183234 | 112 | NA |
| 17 | SC | Soy | 34.159155 | -79.272908 | 118 | 15 |
| 18 | SC | Corn | 34.156593 | -79.27027 | 115 | 4 |
| 19 | NC | Corn | 34.508193 | -78.70899 | 106 | 8 |
| 20 | TN | Corn | 35.830692 | -85.777871 | 61 | 13 |
| 21 | NC | Soy | 35.369816 | -77.877314 | 109 | 11 |
| 22 | NC | Corn | 36.1436 | -78.053422 | 107 | NA |
| 23 | TN | Corn | 35.067905 | -86.62955 | 110 | **34** |
| 25 | NC | Corn | 34.616361 | -79.051667 | 100 | NA |
| 26 | TN | Soy | 35.533413 | -85.951902 | 115 | 12 |
| 28 | SC | Corn | 34.097917 | -80.377715 | 117 | 18 |
| 29 | NC | Corn | 34.705135 | -78.738897 | 112 | **19** |
| 30 | TN | Corn | 35.31105 | -85.945003 | 115 | 15 |
| 31 | TN | Corn | 35.608482 | -85.846379 | 105 | 16 |
| 32 | TN | Corn | 35.099356 | -86.225509 | 75 | 12 |
| 33 | OH | Corn | 39.858763 | -83.669821 | 112 | 7 |
| 34 | OH | Corn | 39.44316 | -83.910189 | 117 | 8 |
| 35 | IN | Corn | 39.853945 | -85.770156 | 120 | **24** |
| 36 | IN | Corn | 40.565608 | -85.503826 | 118 | **28** |
| 37 | OH | Corn | 39.583755 | -83.758264 | 115 | NA |
| 38 | OH | Soy | 39.515447 | -83.407431 | 118 | **25** |
| 39 | IN | Corn | 39.988984 | -85.742262 | 113 | **23** |
| 40 | OH | Soy | 41.284684 | -83.847252 | 117 | 16 |
| 41 | VA | Corn | 38.636343 | -78.472921 | 116 | **23** |
| 42 | VA | Corn | 38.373523 | -78.662516 | 119 | **25** |
| 43 | VA | Soy | 36.886448 | -78.553156 | 67 | **26** |
| 44 | VA | Soy | 38.285415 | -78.797088 | 113 | 14 |
| 45 | VA | Soy | 36.847945 | -78.595042 | 115 | NA |
| 46 | TN | Soy | 35.536019 | -86.17985 | 61 | 12 |
| 47 | SC | Soy | 34.282132 | -79.746597 | 116 | NA |
| 48 | TN | Corn | 35.31653 | -87.35373 | 114 | NA |
| 51 | TN | Soy | 35.533413 | -85.951902 | 94 | NA |
| 52 | OH | Corn | 41.284684 | -83.847252 | 116 | NA |
| 53 | OH | Corn | 39.735642 | -85.763957 | NA | 7 |
| 54 | OH | Corn | 40.255337 | -85.548739 | NA | 10 |

**Table S2** Number of individuals used for each population and treatment combination. Shown are counts of individuals over 2 replicate experiments that were used across populations for each treatment (0-3.4 kg a.i./ha dose of glyphosate) and total individuals used across treatments.

|  |  |  | Dose (kg a.i./ha) | | |  |  |  |  |  |
| --- | --- | --- | --- | --- | --- | --- | --- | --- | --- | --- |
| Population | 0 | 0.21 | 0.42 | 0.84 | 1.7 | | 3.4 | Exp 1 | Exp 2 | Total |
| 1 | 11 | 10 | 10 | 11 | 10 | | 10 | 2 | 60 | 62 |
| 2 | 14 | 17 | 17 | 18 | 17 | | 18 | 41 | 60 | 101 |
| 4 | 20 | 19 | 19 | 18 | 19 | | 20 | 55 | 60 | 115 |
| 5 | 13 | 12 | 10 | 12 | 11 | | 11 | 9 | 60 | 69 |
| 8 | 18 | 16 | 17 | 19 | 17 | | 20 | 49 | 58 | 107 |
| 9 | 15 | 13 | 15 | 16 | 13 | | 15 | 27 | 60 | 87 |
| 10 | 12 | 11 | 13 | 12 | 10 | | 10 | 10 | 58 | 68 |
| 11 | 20 | 20 | 19 | 20 | 17 | | 20 | 57 | 59 | 116 |
| 12 | 19 | 19 | 19 | 20 | 20 | | 20 | 57 | 60 | 117 |
| 14 | 18 | 20 | 20 | 20 | 20 | | 18 | 57 | 59 | 116 |
| 15 | 20 | 20 | 20 | 20 | 19 | | 19 | 58 | 60 | 118 |
| 16 | 18 | 19 | 18 | 20 | 19 | | 18 | 52 | 60 | 112 |
| 17 | 20 | 20 | 20 | 20 | 20 | | 18 | 58 | 60 | 118 |
| 18 | 19 | 20 | 20 | 18 | 18 | | 20 | 55 | 60 | 115 |
| 19 | 17 | 19 | 18 | 17 | 18 | | 17 | 46 | 60 | 106 |
| 20 | 10 | 10 | 10 | 10 | 10 | | 11 | 1 | 60 | 61 |
| 21 | 18 | 18 | 19 | 18 | 16 | | 20 | 51 | 58 | 109 |
| 22 | 17 | 18 | 17 | 17 | 19 | | 19 | 47 | 60 | 107 |
| 23 | 19 | 20 | 17 | 18 | 18 | | 18 | 50 | 60 | 110 |
| 25 | 18 | 16 | 17 | 17 | 16 | | 16 | 40 | 60 | 100 |
| 26 | 18 | 20 | 20 | 19 | 19 | | 19 | 55 | 60 | 115 |
| 28 | 19 | 20 | 19 | 20 | 19 | | 20 | 58 | 59 | 117 |
| 29 | 18 | 19 | 18 | 20 | 18 | | 19 | 52 | 60 | 112 |
| 30 | 20 | 18 | 20 | 19 | 19 | | 19 | 56 | 59 | 115 |
| 31 | 17 | 19 | 20 | 16 | 15 | | 18 | 45 | 60 | 105 |
| 32 | 11 | 11 | 13 | 13 | 12 | | 15 | 15 | 60 | 75 |
| 33 | 19 | 19 | 20 | 17 | 18 | | 19 | 53 | 59 | 112 |
| 34 | 20 | 20 | 19 | 20 | 18 | | 20 | 57 | 60 | 117 |
| 35 | 20 | 20 | 21 | 20 | 19 | | 20 | 59 | 61 | 120 |
| 36 | 18 | 20 | 20 | 20 | 20 | | 20 | 58 | 60 | 118 |
| 37 | 20 | 19 | 20 | 19 | 17 | | 20 | 55 | 60 | 115 |
| 38 | 20 | 20 | 20 | 20 | 18 | | 20 | 58 | 60 | 118 |
| 39 | 20 | 19 | 19 | 18 | 17 | | 20 | 54 | 59 | 113 |
| 40 | 20 | 20 | 20 | 19 | 19 | | 19 | 57 | 60 | 117 |
| 41 | 19 | 19 | 20 | 20 | 18 | | 20 | 57 | 59 | 116 |
| 42 | 20 | 19 | 20 | 20 | 20 | | 20 | 60 | 59 | 119 |
| 43 | 10 | 11 | 13 | 11 | 11 | | 11 | 7 | 60 | 67 |
| 44 | 18 | 20 | 20 | 20 | 16 | | 19 | 55 | 58 | 113 |
| 45 | 19 | 20 | 19 | 20 | 17 | | 20 | 56 | 59 | 115 |
| 46 | 9 | 10 | 10 | 11 | 11 | | 10 | 2 | 59 | 61 |
| 47 | 19 | 20 | 20 | 19 | 19 | | 19 | 56 | 60 | 116 |
| 48 | 20 | 19 | 19 | 20 | 17 | | 19 | 55 | 59 | 114 |
| 51 | 16 | 17 | 16 | 16 | 14 | | 15 | 35 | 59 | 94 |
| 52 | 19 | 19 | 20 | 20 | 18 | | 20 | 58 | 58 | 116 |
| Total | 804 | 813 | 820 | 817 | 776 | | 818 | 1995 | 2619 | 4614 |

**Table S3** Description of Simple Sequence Repeat loci developed in Kuester et al. (2012). Shown are the locus identification number (Locus ID), forward and reverse primer sequence, dye label on forward primer (Dye Set), melting temperature (TA) repeat motif, number of alleles (NA), percent missing data (% Missing Data), allele size range found in the species, observed (HO) and expected (HE) heterozygosity, FST and associated standard error (SE) and the GenBank accession number (Accession No) for each locus and over all loci (where applicable).

| Locus ID | Forward Primer | Reverse Primer | Dye Set | T_A_ | Repeat Motif | N_a_ | % Missing Data | Allele Size Range | H_O_ | H_E_ | F_ST_ | SE | Accession No |
| --- | --- | --- | --- | --- | --- | --- | --- | --- | --- | --- | --- | --- | --- |
|  |  |  |  |  |  |  |  |  |  |  |  |  |  |
| IP1 | CAATTTTTGCAATTCCTTCTACCG | TGTTTGGTTGGTTAAGGAACATGA | 6FAm | 57 | ATA | 2 | 25 | 260-263 | 0.2 | 0.32 | 0.037 | 0.023 | KC122221 |
| IP2 | GCAAAAACGAGAAGCTAGCACAAT | GCTTCAGTAGAGAGCGTTAATGGC | Vic | 57 | ATA | 4 | 11 | 90-115 | 0.4 | 0.53 | 0.291 | 0.235 | KC122206 |
| IP6 | GCTTGATTGATGCAGAATGGTTT | TCAAAATCAAAGAAATCAAATCTCAGAC | Vic | 57 | ATT | 3 | 9 | 176-191 | 0.15 | 0.13 | 0.01 | 0.008 | KC122224 |
| IP8 | ATGCTTTCATTCCCAACTCCATT | AAGGAATGCAAAAAGCTTCATTTG | Vic | 57 | TAT | 4 | 23 | 130-161 | 0.31 | 0.27 | 0.2 | 0.055 | KC122225 |
| IP12 | GTTTTTAACCCACCCACAAGATTT | AGCATGCTTGTCTTTGTTGTTTACT | 6Fam | 57 | AAT | 3 | 18 | 166-169 | 0.04 | 0.04 | 0.021 | 0.01 | KC122208 |
| IP18 | TGGGGCATAGGAAACCAGATTTA | GGACCCAATTGTTCACACTATTGAC | Vic | 57 | AGG(1)ATT(1) | 4 | 25 | 194-206 | 0.12 | 0.27 | 0.069 | 0.02 | KC122223 |
| IP21 | CATCAAATTAAAGCTTCAAACAAAGG | AAGACATAAATGCCCTTGTACAAAAA | Pet | 57 | ATA(1)ATG(1) | 5 | 16 | 173-194 | 0.56 | 0.48 | 0.255 | 0.045 | KC122209 |
| IP26 | TTTATCCACTTTTGTTGCCTTTTTG | TAAGGGAGCAACCCAATACCAGT | 6Fam | 57 | ATT | 3 | 18 | 243-252 | 0.05 | 0.06 | 0.052 | 0.015 | KC122211 |
| IP27 | CATTCCAGCTCCATACGGATACC | CTAGCGGAACCAGTTCTACCATTG | Pet | 57 | CAG | 5 | 21 | 148-169 | 0.21 | 0.37 | 0.103 | 0.023 | KC122212 |
| IP31 | GGAAGCAAAGATCCAAACATGTAA | TGTGTCATACCAAGAATAGTTGTCTTTT | 6Fam | 57 | GA | 2 | 3 | 92-104 | 0.87 | 0.48 | 0.081 | 0.019 | KC122213 |
| IP34 | ACCGCTGGTTCTAACCGTGAATTA | AATTTTGTAGGGTCCACAAATGGG | 6Fam | 57 | TAT | 2 | 7 | 162-165 | 0 | 0.01 | 0.016 | 0.006 | KC122214 |
| IP36 | CAATATTGAAATGAAATGTGAGGTAGTG | TCCACATATTTCTGCACATACACC | 6Fam | 57 | TGTGAA | 2 | 0 | 90-96 | 0.76 | 0.46 | 0.061 | 0.019 | KC122215 |
| IP42 | TGATCCTAAATACAAGGACATTTTGA | TGGGTCTTAGATTTATATTTGCCG | Pet | 57 | TTTTCT | 3 | 24 | 132-142 | 0.31 | 0.26 | 0.17 | 0.042 | KC122217 |
| IP45 | GTCAATTCATGTTCTGAGTTGAAAAA | TTTGATTTTGAATCGTTGGTTCAT | Ned | 57 | AAAAT | 2 | 21 | 165-170 | 0.25 | 0.34 | 0.218 | 0.072 | KC122219 |
| IP47 | TGATTTTCCTCTTCAGCCATGATT | TCTGAATGTATCACTGCAAGGTTTTC | Vic | 57 | AAAAT | 2 | 14 | 123-128 | 0.18 | 0.3 | 0.276 | 0.054 | KC122220 |
| All |  |  |  |  |  |  | 16 |  | 0.29 | 0.29 | 0.126 | 0.029 |  |

**Table S4** Parameters used in the DIYABC analysis. Shown are the minimum and maximum value range for each parameter and associated units. Also indicated were relationships between time parameters that were specified in the model.

| Parameter | Min | Max | Units |
| --- | --- | --- | --- |
| Ne | 250 | 1000 | individuals |
| N1 | 25 | 100 | individuals |
| t1 | 180 | 210 | generations |
| t2 | 180 | 210 | generations |
| t3 | 38 | 44 | generations |
| t4 | 38 | 180 | generations |
| t5 | 2 | 38 | generations |
| Relationships |  |  |  |
| t1>t2 |  |  |  |
| t5<t2 |  |  |  |
| t4<t1 |  |  |  |

**Table S5.** The effective dose across *I. purpurea’s* range. Shown are estimates of the effective dose that eliminates 50% of the population and standard error (SE) for the species mean, Midwestern and Southeastern US regions and each of the 6 sampled states.

| Area | ED50 value | SE |
| --- | --- | --- |
| Species | 1.6 | 0.25 |
| Midwest | 1.04 | 0.44 |
| Southeast | 1.78 | 0.74 |
| IN | 0.61 | 0.22 |
| OH | 1.43 | 0.84 |
| SC | 0.64 | 0.17 |
| NC | 3.1 | 5.29 |
| TN | 2.58 | 2.95 |
| VA | 1.9 | 2.09 |

**Table S6** Descriptive statistics per site across multilocus genotypes. Shown are site location, number of alleles (NA), effective number of alleles (Ne), allelic richness (AR), the expected and observed heterozygosity (He and Ho, respectively), and inbreeding coefficient (F). The bolded value indicates an inbreeding coefficient significantly greater than zero.

| Population | NA | Ne | AR | H_e_ | H_o_ | F |
| --- | --- | --- | --- | --- | --- | --- |
| 2 | 2.07 | 1.57 | 1.31 | 0.307 | 0.316 | -0.007 |
| 4 | 2 | 1.57 | 1.3 | 0.304 | 0.269 | 0.083 |
| 5 | 2.07 | 1.47 | 1.23 | 0.23 | 0.265 | -0.051 |
| 8 | 2.07 | 1.55 | 1.3 | 0.302 | 0.239 | 0.142 |
| 10 | 2.13 | 1.63 | 1.35 | 0.347 | 0.292 | 0.147 |
| 11 | 1.6 | 1.57 | 1.28 | 0.281 | 0.293 | -0.246 |
| 12 | 2.07 | 1.51 | 1.28 | 0.282 | 0.255 | 0.094 |
| 14 | 2 | 1.5 | 1.28 | 0.28 | 0.248 | 0.141 |
| 15 | 2.13 | 1.53 | 1.3 | 0.303 | 0.253 | 0.143 |
| 17 | 1.93 | 1.65 | 1.33 | 0.329 | 0.289 | 0.076 |
| 19 | 1.93 | 1.62 | 1.34 | 0.339 | 0.334 | -0.662 |
| 20 | 1.87 | 1.49 | 1.26 | 0.255 | 0.305 | -0.105 |
| 21 | 1.87 | 1.46 | 1.23 | 0.234 | 0.285 | -0.208 |
| 23 | 2.13 | 1.58 | 1.32 | 0.319 | 0.307 | -0.122 |
| 26 | 1.87 | 1.53 | 1.27 | 0.273 | 0.322 | 0.036 |
| 28 | 2.07 | 1.62 | 1.32 | 0.318 | 0.281 | -0.227 |
| 29 | 2 | 1.6 | 1.31 | 0.312 | 0.282 | 0.101 |
| 30 | 2.2 | 1.58 | 1.31 | 0.309 | 0.293 | 0.117 |
| 31 | 2.07 | 1.75 | 1.37 | 0.372 | 0.384 | 0.057 |
| 32 | 1.73 | 1.47 | 1.25 | 0.251 | 0.261 | -0.05 |
| 33 | 1.8 | 1.66 | 1.33 | 0.326 | 0.332 | -0.078 |
| 34 | 1.87 | 1.7 | 1.33 | 0.33 | 0.28 | -0.05 |
| 35 | 2.27 | 1.63 | 1.33 | 0.332 | 0.273 | 0.122 |
| 36 | 2 | 1.59 | 1.3 | 0.303 | 0.316 | 0.152 |
| 38 | 2.2 | 1.58 | 1.31 | 0.31 | 0.307 | 0.043 |
| 39 | 2.13 | 1.61 | 1.32 | 0.319 | 0.373 | 0.067 |
| 40 | 1.87 | 1.49 | 1.26 | 0.26 | 0.358 | -0.131 |
| 41 | 2.2 | 1.63 | 1.36 | 0.358 | 0.29 | -0.29 |
| 42 | 2.13 | 1.4 | 1.25 | 0.246 | 0.191 | 0.155 |
| 43 | 1.93 | 1.47 | 1.28 | 0.282 | 0.223 | **0.228** |
| 44 | 1.87 | 1.75 | 1.37 | 0.371 | 0.4 | 0.182 |
| 46 | 1.93 | 1.56 | 1.28 | 0.283 | 0.308 | -0.122 |
| 53 | 2 | 1.66 | 1.33 | 0.327 | 0.26 | -0.084 |
| 54 | 1.93 | 1.66 | 1.32 | 0.324 | 0.319 | 0.084 |
| Overall | 2 | 1.58 | 1.3 | 0.304 | 0.294 | 0.008 |

**Table S7.** Analysis of Molecular Variance (AMOVA) of neutral genetic data for which we had at least 18 individuals per population (15 populations, bolded in Table S1). Shown are the main effects of Region (Midwestern and Southeastern US), State, Population and Individual, F-statistic, and F and P values.

| Effect | F-statistic | F | P |
| --- | --- | --- | --- |
| Region | F_RT_ | 0.038 | 0.001 |
| State (Region) | F_SR_ | 0.090 | 0.001 |
| Population (State) | F_PS_ | 0.124 | 0.001 |
| Individual | F_IT_ | 0.510 | 0.001 |

**Table S8.** Description of Simple Sequence Repeat loci developed in Kuester et al. (2012). Shown are the locus identification number (Locus ID), number of alleles (N_A_), observed (H_O_) and expected (H_E_) heterozygosity, F_ST_ and associated standard error (SE) for each locus and over all loci (where applicable).

| Locus ID | N_A_ | H_O_ | H_E_ | F_ST_ | SE |
| --- | --- | --- | --- | --- | --- |
| IP1 | 2 | 0.189541 | 0.379842 | 0.117 | 0.055 |
| IP2 | 3 | 0.359258 | 0.533963 | 0.035 | 0.015 |
| IP6 | 2 | 0.14422 | 0.126517 | 0.077 | 0.03 |
| IP8 | 2 | 0.264281 | 0.254052 | 0.051 | 0.028 |
| IP12 | 1 | 0.057579 | 0.058442 | 0.048 | 0.019 |
| IP18 | 2 | 0.150971 | 0.293732 | 0.08 | 0.031 |
| IP21 | 3 | 0.589956 | 0.487198 | 0.081 | 0.025 |
| IP26 | 2 | 0.051451 | 0.061519 | 0.022 | 0.018 |
| IP27 | 2 | 0.147213 | 0.35362 | 0.232 | 0.084 |
| IP31 | 2 | 0.835285 | 0.476414 | 0.013 | 0.006 |
| IP34 | 1 | 0 | 0.008571 | 0.117 | 0.057 |
| IP36 | 2 | 0.736193 | 0.459591 | 0.019 | 0.015 |
| IP42 | 3 | 0.334493 | 0.301939 | 0.012 | 0.009 |
| IP45 | 2 | 0.248076 | 0.375992 | 0.185 | 0.061 |
| IP47 | 2 | 0.2043 | 0.332607 | 0.244 | 0.053 |
| All |  | 0.287521 | 0.300267 | 0.125 | 0.022 |

**Table S9.** Likelihood values (Ln(P(D)) from STRUCTURE analysis with only populations where sample size was greater than 18 individuals per population.

|  | Number of Populations | | | | | | | | | | | | | | |
| --- | --- | --- | --- | --- | --- | --- | --- | --- | --- | --- | --- | --- | --- | --- | --- |
| Run | 1 | 2 | 3 | 4 | 5 | 6 | 7 | 8 | 9 | 10 | 11 | 12 | 13 | 14 | 15 |
| 1 | -5562.1 | -5294.0 | -5079.8 | -4933.5 | -4819.3 | -4738.4 | -4671.7 | -4628.4 | -4573.6 | -4528.8 | -4498.6 | -4473.1 | -4446.0 | -4431.8 | -4432.6 |
| 2 | -5562.1 | -5294.2 | -5080.0 | -4934.0 | -4819.5 | -4740.1 | -4665.3 | -4617.8 | -4573.3 | -4533.7 | -4495.0 | -4467.3 | -4447.4 | -4437.6 | -4432.8 |
| 3 | -5562.1 | -5294.4 | -5080.3 | -4932.8 | -4818.9 | -4739.5 | -4663.7 | -4618.3 | -4573.5 | -4529.3 | -4496.4 | -4465.2 | -4442.8 | -4436.0 | -4428.7 |
| 4 | -5562.1 | -5294.5 | -5078.9 | -4933.2 | -4821.3 | -4738.5 | -4664.4 | -4633.8 | -4571.5 | -4529.4 | -4495.3 | -4466.1 | -4445.8 | -4430.8 | -4434.5 |
| 5 | -5562.1 | -5294.7 | -5078.6 | -4934.0 | -4821.3 | -4738.3 | -4661.2 | -4618.0 | -4573.5 | -4530.6 | -4494.2 | -4467.2 | -4448.8 | -4434.4 | -4466.0 |
| Average | -5562.1 | -5294.4 | -5079.5 | -4933.5 | -4820.1 | -4739.0 | -4665.3 | -4623.3 | -4573.1 | -4530.4 | -4495.9 | -4467.8 | -4446.2 | -4434.1 | -4438.9 |
| Standard Deviation | 0.00 | 0.27 | 0.73 | 0.52 | 1.15 | 0.80 | 3.91 | 7.41 | 0.89 | 1.98 | 1.70 | 3.10 | 2.23 | 2.83 | 15.29 |

**Figure S1**. STRUCTURE assignment of individuals to two genetic clusters. Small bars represent the assignment of individuals to clusters, with sampling locations differentiated by thick black lines for 15 populations sampled with sample sizes greater than 18 individuals. Shown are each population denoted state (IN, OH, NC, SC, TN, VA) and population ID number.
